# Exploring the differences in serum metabolite profiles after intake of red meat in women with rheumatoid arthritis and a matched control group

**DOI:** 10.1007/s00394-023-03257-y

**Published:** 2023-10-09

**Authors:** Helen M. Lindqvist, Inger Gjertsson, Erik Hulander, Linnea Bärebring, Anna Winkvist

**Affiliations:** 1 Department of Internal Medicine and Clinical Nutrition, Institute of Medicine, Sahlgrenska Academy, University of Gothenburg, Gothenburg, Sweden; 2 Department of Rheumatology and Inflammation Research, Institute of Medicine, Sahlgrenska Academy, University of Gothenburg, Gothenburg, Sweden; 3 Department of Rheumatology, Sahlgrenska University Hospital, Gothenburg, Sweden

**Keywords:** Rheumatoid arthritis, Metabolomics, Postprandial, Red meat

## Abstract

**Purpose:**

Studies have suggested that women with RA tend to avoid red meat more often than women without RA, based on their perception that it exacerbates their symptoms. Therefore, the aim of this study is to investigate and compare the postprandial metabolic response following the consumption of a red meat meal in patients with RA and a matched control group.

**Methods:**

Participants were challenged with a meal with red meat and blood samples were collected before and at 0.5, 1, 2, 3 and 5 h after the meal. Serum metabolites were quantified by Nuclear Magnetic Resonance (NMR) analysis. Orthogonal Projections to Latent Structures with Discriminant Analysis (OPLS-DA) was used to evaluate separation by metabolites due to diagnosis of RA or not and to identify changes in metabolites related to RA. Incremental area under the curve was calculated for univariate comparisons for 23 metabolites.

**Results:**

The matched groups, including 22 women with RA and 22 women without RA, did not differ significantly in age, body mass index, diet quality or reported physical activity. OPLS-DA models had a limited quality indicating that there were no differences in metabolite patterns between the groups. However, phenylalanine was significantly higher in concentration in women with RA compared to controls in both fasting and postprandial samples.

**Conclusion:**

To conclude, this well-controlled postprandial intervention study found a significantly higher concentration of phenylalanine in both fasting and postprandial samples of women with RA compared to matched women without RA. These findings warrant further investigation in larger studies.

**Trial registration:**

The PIRA (Postprandial Inflammation in Rheumatoid Arthritis) trial is Registered at Clinicaltrials.gov (NCT04247009).

**Supplementary Information:**

The online version contains supplementary material available at 10.1007/s00394-023-03257-y.

## Introduction

Rheumatoid arthritis (RA) is a chronic autoimmune disorder characterized by persistent synovitis, joint destruction, pain, and systemic inflammation. It affects around 1% of the world's population, negatively impacting joint mobility and quality of life. Anti-rheumatic therapies have improved in recent decades, but pain, disability, and fatigue persist in many patients [1–3]. Thus, additional treatment is warranted, including lifestyle interventions. In addition, it is known that diet is strongly associated with other chronic diseases, such as cardiovascular disease, cancer, and diabetes, but efforts to provide dietary treatment for patients with RA are hampered by the limited evidence. To determine if existing evidence from other populations is relevant also in RA, it is of interest to establish if effects from different foods and diets are similar in persons with and without RA. It has, for example, been reported that women with RA specifically avoid red meat to a greater extent than women without RA [4, 5], due to the belief that it aggravates their symptoms [6]. The reason as to why red meat would impact disease activity is unknown. It has been speculated that red meat could contribute to increased inflammation, but there is no clear evidence for this, mainly because of a limited number of studies [7, 8]. A review concluded that studies on diets low in red meat (Mediterranean, vegetarian, vegan) showed reduction in pain, but with a low strength of evidence [9].

A first step to study a different reaction to red meat between persons with and without RA could be a controlled meal study. In fact, postprandial lipid concentrations have been shown to be more predictive of future disease development than those taken in the fasted state [10]. The postprandial state may also be more informative of the inflammation response after dietary exposure than samples collected in the fasting state [11]. Despite this little is known on how different diseases affect the response to diet postprandially. Studies have shown differences in fasting levels of metabolites between patients with RA and healthy controls [12–17]. Unfortunately, these studies have often neglected basic characteristics that could impact the metabolome, such as age, sex, body mass index, diet, and menopausal status which obstruct the interpretation of the results. Only Li et al. [18] reported data on these parameters, which did not differ significantly between the patients with RA and the control group. The authors found that several metabolites differed between the two groups, including amino acids such as leucine, phenylalanine, and proline. However, if the postprandial response to single meals is altered in RA is not known.

## Methods

### Aim

The objective of this study was to investigate if women with RA respond differently than a matched group of women without RA to a meal with red meat, reflected in the postprandial serum metabolome.

### Study design

The study had a parallel single meal design. Samples were collected before the meal and at 30 min, one, two, three and five hours post ingestion of the meal. Women with RA were matched on age and body mass index (BMI) on group basis with women without RA.

### Participants

Women with diagnosis of RA (International Classification of Diseases-code M05.9 or M05.8) in the age span 20–70 years listed at the Sahlgrenska University Hospital were identified through the Swedish Rheumatology Quality Register. Potential participants (*n* = 934) were contacted by letter. Women in the same age span without diagnosis for RA or other rheumatic diseases were recruited as controls by advertisement in social media, by word of mouth and by posters at official noticeboards. Inclusion criteria for patients with RA were ≥ 2 years since RA diagnosis and no changes in disease modifying anti-rheumatic drugs (DMARDs) the past 3 months. Exclusion criteria were underweight (BMI < 18.5 kg/m^2^) or obesity (BMI ≥ 30 kg/m^2^), diagnosis of cancer, diabetes, inflammatory bowel disease, celiac disease, allergy or intolerance to any of the foods served in the study, pregnancy, lactation, use of any lipid lowering medication, glucocorticoids or interleukin-6 (IL-6) inhibiting therapy during the past 4 weeks, smoking, hemoglobin levels ≤ 100 g/L, glycated hemoglobin (HbA1c) above reference range (18–50 years: > 42 mmol/mol and > 50 years: > 46 mmol/mol).

The PIRA (Postprandial Inflammation in Rheumatoid Arthritis) trial is Registered at Clinicaltrials.gov (NCT04247009) and was approved by the Swedish Ethical Review Authority (Dnr 2019-05242).

### Sample collection and analysis

C-reactive protein (CRP), erythrocyte sediment rate (ESR), hemoglobin and HbA1c were measured in fresh samples according to the clinical routine at the Sahlgrenska University Hospital laboratory. Tender and swollen joints were examined by trained nurses at the Department of Clinical Rheumatology Research Center at the Sahlgrenska University Hospital. The patients filled out visual analogue scales (VAS) for global health, pain, and fatigue. Disease Activity Score 28-joints (DAS28) was calculated with erythrocyte sedimentation rate [19]. The participants’ medications were controlled through interviews and from patient records, and those fulfilling any of the exclusion criteria were excluded.

Physical activity was assessed based on scales between 1 and 5 on habitual physical activity and intentional physical exercise. Based on this, a physical activity index between 1 and 4 was calculated, resembling that previously validated by Wareham et al. [20]. Dietary quality index was assessed based upon food frequency questionnaires, whereby an index ranging from 1 to 12 was constructed, as previously described by the Swedish Food Agency, to assess habitual quality of diet [21].

### Intervention meal

The intervention meal consisted of two hamburgers. The burgers contained 130g minced meat (Produced by Scan, by Swedish meat products, 60% beef, 40% pork), 25 g egg, 8 g breadcrumbs and served with 84 g (2 slices) toasted white bread (Jättefranska, Pågen AB), 10 g (2 leaves) romaine lettuce, 10–20 g (4 slices) cucumber, 20–30 g (2 slices) tomato and 20 g vegan hamburger dressing (Hamburger dressing 250 mL, Rydbergs AB). All burgers were cooked to mid-temperature 75̊ °C in non-stick pans with 1 teaspoon of canola oil.

The burgers were cooked in two batches (December 2019, August 2021), due to the Covid-19 pandemic that halted the PIRA trial for 18 months. The differences in macronutrients between these two batches of meat burgers were negligible. Protein and fat content were analyzed by Eurofins Food & Feed Testing, Sweden. Carbohydrate content was calculated based on food labels; fiber content not included. This meal contained 35 g protein, 40 g fat, 47 g carbohydrates and about 700 kcal.

### Outcomes

The main outcome in this study report was differences in postprandial metabolite pattern at 3 h after the study meal, between women with and without RA. Secondary outcomes were differences in metabolite patterns in the fasting state and other postprandial timepoints as well as differences in incremental area under the curve (AUC_min_) for quantified metabolites.

### Blood sampling and preparation

During the postprandial meal challenges, a catheter was placed, and serum samples were taken in the fasting state and after 30 min, one, two, three and five hours post ingestion of the meal. Serum was collected in letting tubes (BD Vacutainer, 5 mL, reference no 367624), left for 30 min in room temperature, thereby refrigerated for 30 min before centrifugation for 10 min in 2600*g*. After centrifugation and isolation procedures, all samples were immediately stored in − 20 °C, and at earliest convenience transferred to − 80 °C for storage until analysis.

Metabolites were quantified by Nuclear Magnetic Resonance (NMR)-analysis; serum samples were prepared according to In Vitro Diagnostics Research (IVDr) standard operating procedures (Bruker BioSpin; www.bruker.com/products/mr/nmr/avanceivdr.html). In brief, serum samples were thawed at room temperature for 30 min, then centrifuged at 3500×*g* for 1 min at 4 °C. Thereafter, 325 μl of serum was transferred with a SamplePro L liquid handler (Bruker BioSpin) to a deepwell plate (Porvair, cat. no 53.219030) containing 325 μl NMR buffer ((75 mM sodium phosphate, pH 7.4, 0.08% 3-(trimethylsilyl) propionic-2,2,3,3-d4), 0.04% sodium azide, 20% v/v D2O) per well. The plate was shaken at 400 rotations per minute, 12 °C for 5 min in a Thermomixer Comfort (Eppendorf). Finally, 600 μl sample was transferred to 5 mm SampleJet NMR tubes with the SamplePro L. The sample tubes, deepwell plate and SampleJet rack were kept at 2 °C during the preparation in the SamplePro L robot.H NMR data was acquired on a Bruker 600 MHz Avance III spectrometer equipped with a room temperature 5 mm BBI probe and a cooled SampleJet sample changer. In brief, 1D NOESY (‘noesygppr1d’ pulse sequence), 1D CPMG (‘cpmgpr1d’) and 2D J-resolved (‘jresgpprqf’) spectra were acquired according to the standard IVDr parameter settings at 37 °C. A pre-acquisition temperature stabilization time of 300 s was used. Before measurement, all samples were kept at 6 °C in the SampleJet. Experimental parameters are available upon request. The 1H-NMR spectra were aligned by setting the TSP-d4 to 0 ppm using icoshift and the spectra were bucketed using the function “opt_bucket.m” [22]. This function used initial size of bucket = 0.04 and slackness = 0.5. The 1D NOESY data were also submitted for B.I.-Lisa lipoprotein profiling and B.I.Quant-PS 2.0.0 automatic quantification of a subset of metabolites through a remote secure Bruker server, generating in total 39 B.I.Lisa and 41 B.I.Quant-PS variables. After quality control using Brucker data on significant correlation for each sample and excluding metabolites where more than 50% of the samples had less than 85% significant correlation, 23 quantified serum metabolites remained for analysis. Included quantified metabolites were: trimethylamine-N-oxide, alanine, creatine, creatinine, glutamine, glycine, histidine, isoleucine, leucine, *N*,*N*-dimethylglycine, phenylalanine, tyrosine, valine, acetic acid, citric acid, formic acid, lactic acid, succinic acid, acetoacetic acid (acetoacetate), acetone, pyruvic acid, glucose, and dimethylsulfone.

For quantified metabolites, AUC_min_, i.e., the area above the lowest value of all time points, were calculated. Phenylalanine is converted to tyrosine by phenylalanine (4)-hydroxylase (PAH) and to estimate the enzyme conversion we calculated the phenylalanine/tyrosine ratio as a proxy for PAH activity [23].

### Statistical analysis

#### Multivariable methods

All multivariable analyses were performed using SIMCA software v.17.0 (Umetrics AB, Umeå, Sweden) and no samples were excluded in any of the analysis. 188 buckets within the chemical shift range of − 0.05 to 8.0 ppm, excluding the water peak at 4.5–5.0 ppm, were included in the multivariable analysis.

Principal component analysis (PCA) model was used to explore clustering patterns of observations and trends in the data in relation to known factors and outliers. Separation of classes and variables related to separation in the data according to classification of diagnosis of RA or not were evaluated using an Orthogonal Projections to Latent Structures with Discriminant Analysis (OPLS-DA) in fasting samples and at different time points (variable concentration at time X minus variable concentration at time 0). Cross-validation groups were set to 7 (default). The validity of OPLS-DA models was assessed using permutation tests (*n* = 999). Validated prediction models for performance are presented using Receiver Operating Characteristics (ROC) Curve for OPLS-DA models. Also, cross-validated predictive residuals (CV-ANOVA) visual comparison between scores and cross-validated scores, the cumulative amount of explained variation in the data summarized by the model (R2X[cum] and R2Y[cum]), and the predictive ability of the model (Q2[cum]) are presented. Class discriminating variables of interest from the OPLS-DA models were selected if the model had a significant (*p* < 0.05) CV-ANOVA and the permutation plot showed that the model had a sufficient quality.

#### Univariate methods

Statistical analyses were performed using SPSS version 25 (SPSS Inc., Chicago, IL, USA). Mann–Whitney *U*-test was used since the subjects only were matched on a group level. Univariate tests were performed to compare baseline characteristics and metabolites between women with RA and without RA. Univariate tests were also performed for AUC_min_ and metabolites at different time points if they were found either to have a significant AUC_min_ or driving the separation in OPLS-DA models. In this explorative study, data are presented as median (inter quartile range (IQR)) with significance set at *α* = 0.05, i.e., not corrected for multi testing. In addition, to be able to explore if fasting metabolites were associated to certain health variables spearman rank correlation was performed for all participants for BMI, age, HbA1c, Hb, physical activity, and diet index and for metabolites and disease related variables (VAS, CRP, ESR, tender and swollen joints, and DAS-28) for women with RA. A sensitivity test adjusting for BMI and age was also performed for this analysis. For these additional correlation tests significance was set at *α* = 0.01.

#### Power calculation

The primary objective of the PIRA trial was to measure postprandial levels of IL-6. The sample size was calculated for alpha = 0.05 and 80% power, using data from previous studies [24, 25], which suggested a difference of 1.5 pg/mL in IL-6 could be expected with a standard deviation of 2.0. The study aimed at 30 patients with RA and 30 controls completing the trial. However, a sample size of about 20 participants/group has previously been found sufficient to compare metabolite patterns between patients with RA and controls in fasting samples [13, 17, 26], indicating that this is a sufficient group size.

## Results

### Subjects

**Fig. 1:**
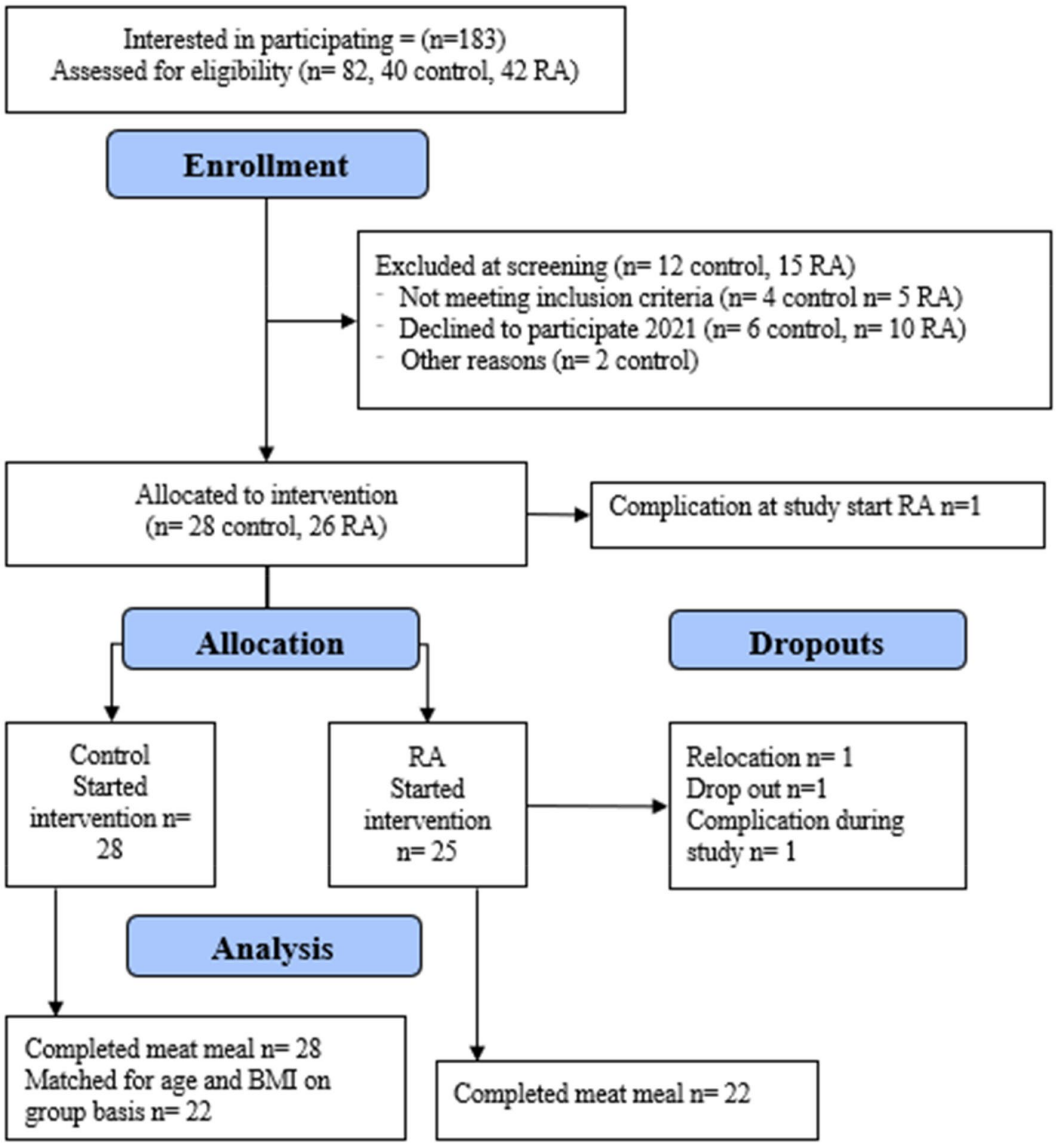
Consolidated standard reporting trial diagram

Recruitment for the PIRA trial started in January 2020. Forty-two patients with RA were interested in participating, fulfilled the pre-screening criteria and were invited to the screening visit (Fig. 1). Due to the COVID-19 pandemic resulting in the study being postponed 1.5 year, ten of these patients declined to participate when the study was resumed in 2021 and five patients did not meet the inclusion criteria. In total, three patients with RA completed their meal in 2020 and 19 in 2021. Forty controls fulfilled the pre-screening criteria and were invited to screening during 2020 and 2021, but six of these declined to participate when the study was resumed in 2021 and additionally two were asked not to participate because it was not any longer possible to match them with patients (e.g., too young, or too low BMI). The pandemic restrictions increased again in November 2021 and limited the possibility to continue recruiting further controls resulting in that patients and controls only could be matched on a group-level. Four controls completed their meal in 2020 and 24 in 2021 and 22 of these were matched to the 22 patients based on age and BMI.

Baseline characteristics are presented in Table 1. The matched groups did not differ significantly in age, BMI, diet quality or reported physical activity. None of the participants had a high-quality diet based on diet index and five of the patients with RA (23%) and three of the controls (14%) had a low-quality diet. HbA1c was higher in the control group and was significantly correlated to glucose (*r* = 0.388, *p* = 0.009). No other metabolites were associated to either of the baseline characteristics for all participants. Among the disease related markers, VAS pain was associated to sarcosine (*r* = 0.551, *p* = 0.008), however when adjusted for age and BMI this association became weaker (*r* = 0.476, *p* = 0.034). The age ranged between 46 and 71 years at the time of the intervention and all but three women in each group had reached menopause. BMI was between 19 and 31 kg/m^2^ and about 40% were overweight (BMI > 25 kg/m^2^). The patients with RA had a median DAS28 of 2.5, corresponding to a disease in remission.

**Table 1:** Baseline characteristics of the participants

Baseline characteristics	RA (*n* = 22)		Controls (*n* = 22)		*p*
	Median (IQR^a^)	Range	Median (IQR^a^)	Range	
Age (y)	66 (59, 69)	46–71	65 (60, 69)	46–71	0.557
Body mass index (kg/m^2^)	25 (23, 27)	19–31	24 (23, 26)	21–31	0.707
Weight (kg)	69 (61, 76)	48–97	70 (64, 75)	52–90	0.778
Waist circumstance	86 (77, 92)	68–102	83 (79, 93)	69–101	0.716
Hip circumstance	101 (96, 106)	91–177	104 (100, 110)	93–118	0.115
Haemoglobin (g/l)	132 (125, 138)	113–154	133 (130, 142)	113–150	0.29
HbA1c (mmol/mol)	32.0 (30.8, 34.0)	21.0–38.0	35.0 (31.8, 37.0)	26.0–40.0	0.008
DAS28 ^b,c^	2.54 (1.94, 3.45)	1.09–4.76			
DAS28-CRP ^c^	2.33 (1.49, 3.2)	1.15–51.43			
Erythrocyte sedimentation rate (mm/1 h)	11.0 (6.0, 22.5)	1.0–41.0			
C-reactive protein (mg/l)	1.25 (0.54, 3.1)	0.25–6.00			
Swollen joints 28 (no)	1.0 (0.0, 5.0)	0–10			
Tender joints 28 (no)	0.5 (0.0, 2.0)	0–6			
VAS global health (mm)	14 (3, 33)	0–62			
VAS Pain (mm)	20 (1, 32)	0–69			
VAS Fatigue (mm)	16 (1, 55)	0–84			
Physical activity index	3 (2, 4)	1–4	2 (2, 3)	1–4	0.076
Diet index	5.0 (4.8, 7.0)	2.0–8.0	6.0 (5.0, 6.0)	3.0–8.0	0.698
**Rheumatic drug treatment:**	***n***	**%**	***n***	**%**	
Analgesic:	5	23%	4	18%	
NSAIDS *n* (%) csDMARDs	3	14%	1	1%	
methotrexate *n* (%)	18	82%			
hydroxychloroquine *n* (%)	3	14%			
bDMARD’s, *n* (%)	9	41%			

*HbA1c* glycated haemoglobin, *DAS28* disease activity score 28, *VAS* visual analogue scale, *DMARD* disease modifying anti-rheumatic drugs, conventional synthetic (cs), biological (b), non-steroid anti-inflammatory drugs (NSAIDs)

^a^Interquartile Range (first quartile, third quartile), ^c^score

### Differences in fasting metabolites between women with and without RA

No differences in metabolite patterns were found in fasting samples when comparing women with and without RA. The only metabolite that was significantly different in univariate tests between the two groups was phenylalanine, that was significantly higher in concentration in women with RA compared to controls (*p* = 0.008).

### Differences in postprandial metabolites between women with and without RA

**Fig. 2:**
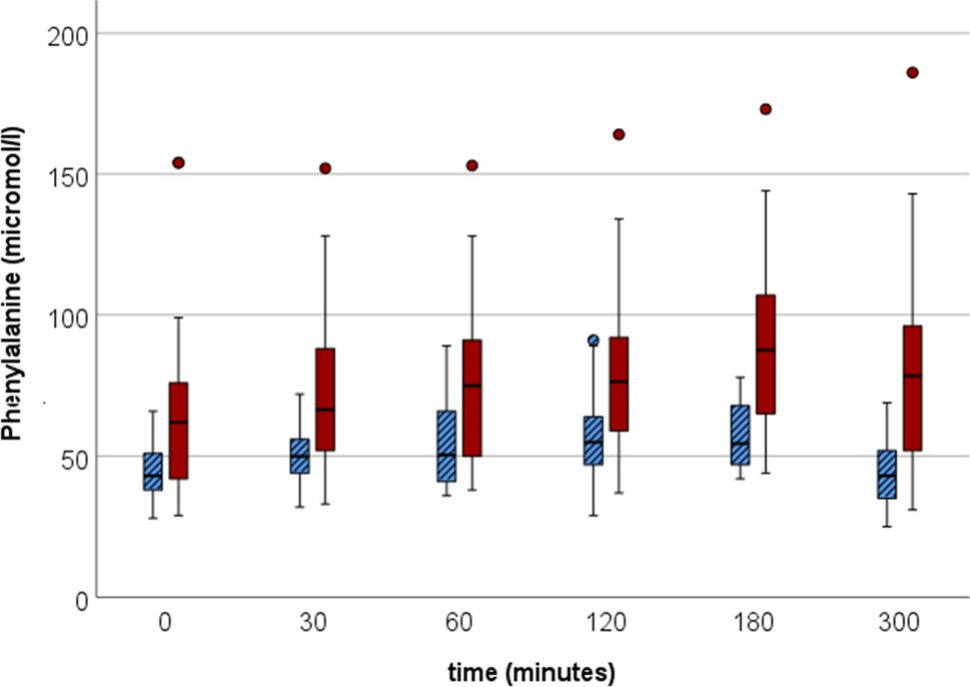
Boxplots of phenylalanine in women with RA (red box) and controls (blue lined box) in fasting and postprandial serum samples. Concentrations of phenylalanine were significantly different at all time points (*p* < 0.05) and for AUC_min_

Postprandial models comparing the groups were possible to create at 1 h, 2 h, 3 h, and 5 h, but none of them had a sufficient quality (Table 2). Due to the limited quality of the models, no metabolites driving the class separations were selected for further scrutiny. In univariate tests of AUC_min_ for the metabolites, again phenylalanine was significantly different between the groups (*p* = 0.007) (Supplementary table 1, Supplementary Fig. 1). Phenylalanine was significantly higher in patients with RA at all timepoints (Fig. 2). In addition, AUC_min_ for acetoacetic acid was significantly lower among women with RA (*p* = 0.046) (Supplementary Fig. 2), but there were no differences at any individual timepoint. In addition, the phenylalanine/tyrosine-ratio was significantly higher among women with RA at all time points. AUC_min_ was most pronounced for both groups for the amino acids alanine, glutamine, glycine, and valine (Supplementary table 1).

**Table 2:** Models statistics for women with and without RA (*n* = 44)

Model	Nr of Lv^1^	R2X [cum]^2^	R2Y [cum]^3^	Q2 [cum]^4^	CV-ANOVA^5^ (p-value)	ROC AUC^6^	Class (RA/K)	Permutation test (Q2)^7^
PCA-X T0 RA + C	3	0.535		0.424				
OPLS-DA RA vs C T0	1 + 2 + 0	0.491	0.747	0.179	0.262	0.99/0.99	95/91	− 0.422
OPLS-DA RA vs C ΔT 0.5 h	1 + 0 + 0	0.439	0.112	0.00444	0.912	0.76/0.76	73/68	− 0.170
OPLS-DA RA vs C ΔT 1 h	0 + 0 + 0							
OPLS-DA RA vs C ΔT 2 h	0 + 0 + 0							
OPLS-DA RA vs C ΔT 3 h	0 + 0 + 0							
OPLS-DA RA vs C ΔT 5 h	1 + 0 + 0	0.423	0.120	− 0.0103	1	0.77/0.77	73/68	− 0.180

1 Latent Variables, 2 Cumulative fraction of the sum of squares of X explained by the selected latent variables, 3 Cumulative fraction of the sum of squares of Y explained by the selected latent variables, 4 Cumulative fraction of the sum of squares of Y predicted by the selected latent variables, estimated by cross validation, 5 ANalysis Of VAriance testing of Cross-Validated predictive residuals, 6 ROC AUC = Receiver Operating Curve Area under curve, 7 The intercept between real and random models, degree of overfit

## Discussion

Previous studies have identified differences in metabolites between patients with RA and healthy controls in fasting samples, but these studies have often failed to account for background characteristics that could affect the metabolome, such as age, sex, BMI, diet, and menopausal status. The current study has taken such factors into consideration by matching the group of RA patients with the control group based on sex, age, and BMI. This is important because it allows for more accurate comparison of the metabolome between the two groups [27], as it is not possible to adjust for these factors in multivariate models such as OPLS-DA. Additionally, data on menopause were collected and there were no significant differences between the two groups.

There are few previous trials evaluating serum metabolites over several hours after a meal of red meat and because of the different ways of presenting these data, comparisons are difficult. However, our finding that alanine, glutamine, and valine had the largest AUC_min_-concentrations, is in line with recent findings from Neacsu et.al. [28]. In our data also AUC_min_ for glycine, was among the higher concentrations, but not isoleucine and leucine.

Interestingly, in this matched comparison, phenylalanine was the only significantly different metabolite between the groups, at baseline and at all postprandial time points. Phenylalanine, among other metabolites, has been reported to differ between patients with RA and healthy individuals in several studies [12, 14, 16, 29–31]. However, Li et al. [18] reported that phenylalanine had the highest specificity (100%) and high sensitivity (86.7%) in ROC analysis of potential biomarkers of RA [18]. In addition, it has been reported that responders to IL-6 inhibitor tocilizumab and to rituximab had higher concentrations of phenylalanine (and some other metabolites) prior to treatment [32, 33], indicating a potential role of phenylalanine in the disease.

The reason for a higher serum phenylalanine concentration among patients with RA is not fully understood, but increased phenylalanine has also been reported in patients with cancer and sepsis [34]. In this work, we also show that the phenylalanine/tyrosin-ratio is higher in patients with RA compared to controls, and this is even more pronounced after intake of a meal containing red meat. This could indicate that the catabolism of phenylalanine is reduced in RA, as a higher phenylalanine/tyrosin-ratio suggests an impaired conversion of phenylalanine to tyrosine by the enzyme phenylalanine-hydroxylase. This could in turn depend on an insufficient supply of cofactor 5,6,7,8-tetrahydrobiopterin (BH4). BH4 is chemically sensitive to oxidation and its’ concentration is also influenced by inflammation [35]. Clinical data show significant reductions in both pain and inflammation in RA and inflammatory bowel disease patients treated with sulfasalazine [36], an inhibitor of sepiapterin reductase [37] leading to reduced BH4 levels. However, it is not possible to draw any conclusions about the mechanisms behind the finding of higher phenylalanine concentrations in women with RA, from our data.

The higher AUC_min_ concentration of acetoacetic acid in women without RA was mainly explained by a non-significantly higher concentration of acetoacetic acid at 5h postprandially. Acetoacetic acid, a ketone body, is a result of fatty acid degradation in the liver. It is transported from the liver by the blood to other cells where it is used for energy in the fasting state [38]. The higher concentration of acetoacetic acid in the current study could perhaps be explained by the fact that the women without RA had higher HbA1c, indicating less glycemic control. Ketone bodies have been reported to be positively associated with HbA1c levels in patients with type 2 diabetes in fasting samples [39]. However, all patients in our study had less than 42 mmol/mol HbA1c in fasting samples and were regarded as non-diabetic or even non-pre-diabetic. Ketone bodies have been reported to both initially induce inflammation, and to also have beneficial effects in the long term [39]. Due to this, in combination with the fact that the difference in AUC_min_ could be a chance finding, we do not want to further speculate as to what caused these results.

Although no other metabolites but phenylalanine differed between the two groups, we found that a higher serum sarcosine concentration was associated to a higher VAS pain. The association was weaker when adjusted for age and BMI. Sarcosine, a biogenic amine involved in methionine, glycine, and folate metabolism, has previously been found to decrease with aging and increase by diet restriction [40]. It is also a competitive inhibitor of glycine type 1 transporter [41], which recently has been pointed out as analgesic targets in inflammatory and chronic pain [42]. Sarcosine has been found to be lower in patients with RA in one study, however BMI for the groups was not provided, and the RA group was slightly older, and it is therefore difficult to draw any conclusions about the serum sarcosine concentrations [43]. Our study shows higher sarcosine in patients with higher reported pain, which is hard to explain, but the there are multiple factors that could influence this result and the association between sarcosine and VAS pain should be confirmed in larger settings prior speculations in this finding.

This study had some limitations; firstly, we failed to match controls to patients at an individual level based on age, BMI, and physical activity. However, the main strength of the PIRA trial is that the study compares patients with RA with a matched group of participants without RA. It is also the first postprandial study in patients with RA studying metabolites, thus adding considerably to the current knowledge based on the nutrition in RA. The meal size of 700 kcal, with a palatable design, is realistic and allows for theorizing about plausible effects of food selection in free-living individuals. However, the main limitation of this study is its small sample size, which limits the generalizability. Additionally, the fact that all participants were women further limits the generalizability of the findings, although most patients with RA are women.

## Conclusions

To conclude, this well-controlled postprandial intervention study found a significantly higher concentration of phenylalanine in both fasting and postprandial samples of women with RA compared to matched women without RA. These findings warrant further investigation in larger studies and indicate that non-fasting samples may be just as useful as fasting samples for identifying discrepancies in phenylalanine concentrations.

### Supplementary Information

Below is the link to the electronic supplementary material.

Supplementary file1 (DOCX 36 KB)

Supplementary file2 (DOCX 37 KB)

Supplementary file3 (XLSX 32 KB)

## Data Availability

The data underlying this article cannot be shared publicly considering the Swedish law on privacy of the individuals involved in the study. However, the data can be shared upon reasonable request to the corresponding author.
