# Supplementary material for: Exploring the differences in serum metabolite profiles after intake of red meat in women with rheumatoid arthritis and a matched control group
Source: Eur J Nutr. 2023 Oct 9;63(1):221–30. doi: 10.1007/s00394-023-03257-y (PMC10798910; doi:10.1007/s00394-023-03257-y)
Supplement: Supplementary file 2 — Supplementary file2 (DOCX 37 KB) [file 394_2023_3257_MOESM2_ESM.docx]

Supplementary figure 2. Area under the curve for acetoacetic acid.


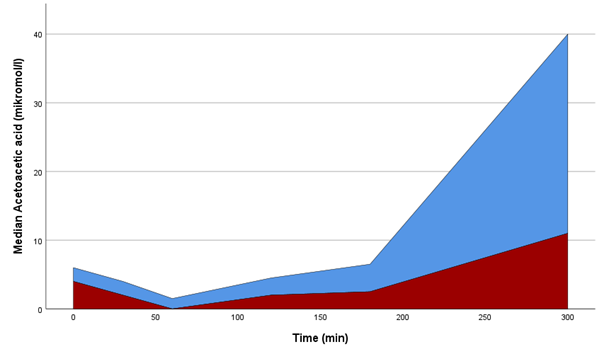


Area under the curve based on median values at each timepoint for acetoacetic acid. Concentrations at each timepoint is calculated by subtracting the minimum value from the concentration at the time for each individual. Women with RA in red and without in blue.
